# Supplementary material for: Associations between Multimorbidity and Physical Performance in Older Chinese Adults
Source: Int J Environ Res Public Health. 2020 Jun 24;17(12):4546. doi: 10.3390/ijerph17124546 (PMC7344642; doi:10.3390/ijerph17124546)
Supplement: Supplementary file 1 [file ijerph-17-04546-s001.pdf]

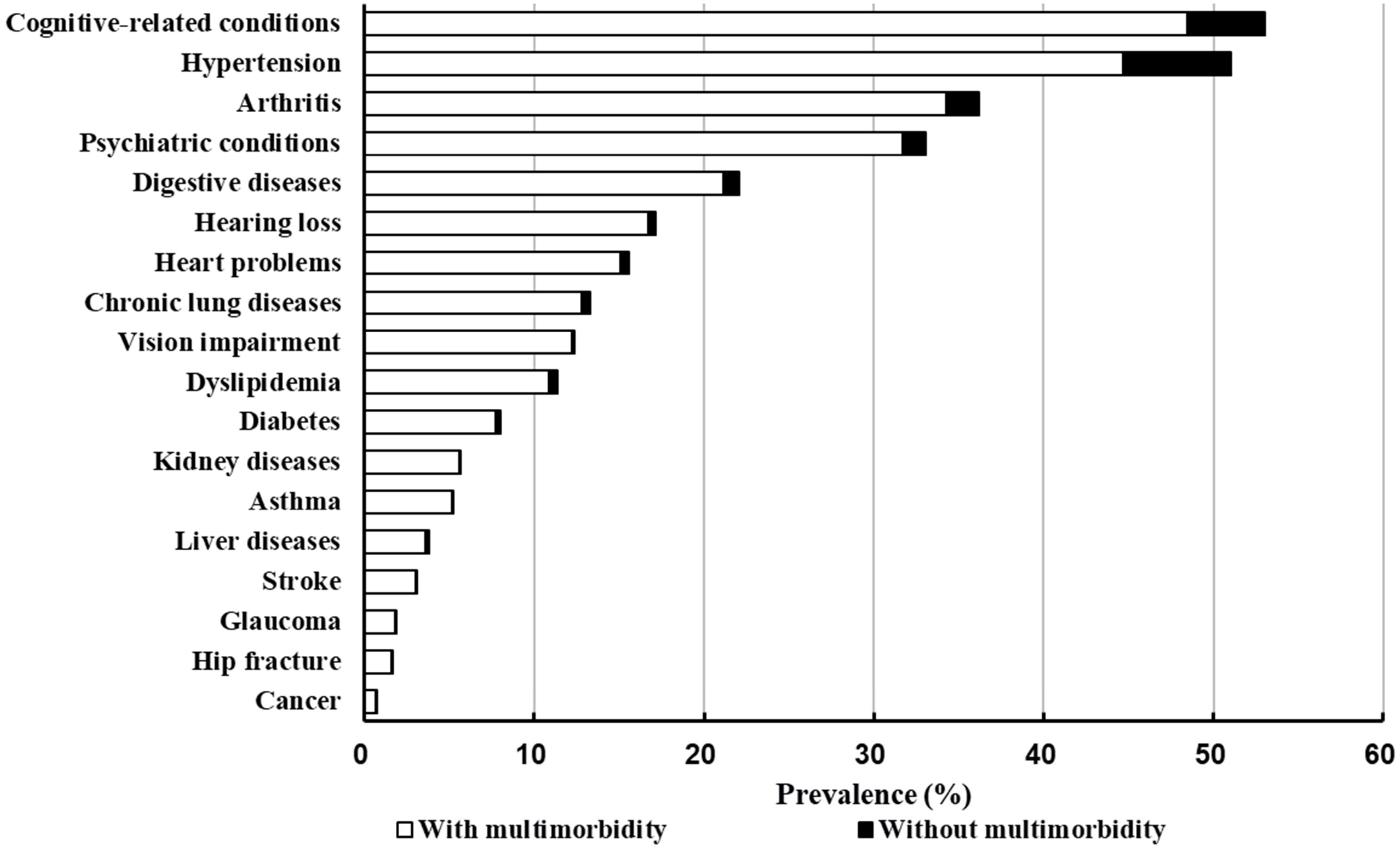

Figure S1. Prevalence of individual chronic conditions at baseline (N = 10,112).

Table S1. Definition of each chronic condition included.

Chronic Conditions	Definition	Chronic Conditions	Definition
Hypertension	Hypertension	Liver diseases	Liver disease (except fatty liver, tumors, and cancer)
Dyslipidemia	Dyslipidemia (elevation of low density lipoprotein, triglycerides (TGs), and total cholesterol, or a low high density lipoprotein level)	Kidney diseases	Kidney disease (except for tumor or cancer)
Diabetes	Diabetes or high blood sugar	Psychiatric conditions	Emotional, nervous, or psychiatric problems or possible depressive symptoms
Heart problems	Heart attack, coronary heart disease, angina, congestive heart failure, or other heart problems	Cognition-related conditions	Memory-related disease (Alzheimer's disease, brain atrophy, Parkinson's disease or other memory-related problems), or possible cognitive impairment, or self-rated poor memory
Stroke	Stroke	Vision impairment	Vision impairment (poor eyesight for seeing things at a distance or up close, even with glasses or corrective lenses if normally wear them)
Chronic lung diseases	Chronic lung diseases, such as chronic bronchitis, emphysema (excluding tumors, or cancer)	Hearing loss	Hearing loss (poor hearing with a hearing aid if normally use it and without if normally don't)
Asthma	Asthma	Glaucoma	Glaucoma
Digestive diseases	Stomach or other digestive disease (except for tumor or cancer)	Hip fracture	Hip fracture
Arthritis	Arthritis or rheumatism	Cancer	Cancer or malignant tumor (excluding minor skin cancers)
